# The Role of MicroRNAs in the Regulation of Gastric Cancer Stem Cells: A Meta-Analysis of the Current Status

**DOI:** 10.3390/jcm8050639

**Published:** 2019-05-09

**Authors:** Vitalba Ruggieri, Sabino Russi, Pietro Zoppoli, Francesco La Rocca, Tiziana Angrisano, Geppino Falco, Giovanni Calice, Simona Laurino

**Affiliations:** 1 Laboratory of Preclinical and Translational Research, IRCCS-CROB, Referral Cancer Center of Basilicata, 85028 Rionero in Vulture, Italy; vitalba.ruggieri@crob.it (V.R.); sabino.russi@crob.it (S.R.); pietro.zoppoli@crob.it (P.Z.); francesco.larocca@crob.it (F.L.R.); giovanni.calice@crob.it (G.C.); 2 Department of Biology, University of Naples Federico II, 80138 Naples, Italy; tiziana.angrisano@unina.it (T.A.); geppino.falco@unina.it (G.F.); 3 Section of Stem Cell and Development, Istituto di Ricerche Genetiche “Gaetano Salvatore” Biogem s.c. a.r.l., 83031 Ariano Irpino, Italy

**Keywords:** gastric cancer, gastric cancer stem cells, self-renewal, miRNAs, meta-analysis

## Abstract

Gastric cancer (GC) remains one of the major causes of cancer-related mortality worldwide. As for other types of cancers, several limitations to the success of current therapeutic GC treatments may be due to cancer drug resistance that leads to tumor recurrence and metastasis. Increasing evidence suggests that cancer stem cells (CSCs) are among the major causative factors of cancer treatment failure. The research of molecular CSC mechanisms and the regulation of their properties have been intensively studied. To date, molecular gastric cancer stem cell (GCSC) characterization remains largely incomplete. Among the GCSC-targeting approaches to overcome tumor progression, recent studies have focused their attention on microRNA (miRNA). The miRNAs are short non-coding RNAs which play an important role in the regulation of numerous cellular processes through the modulation of their target gene expression. In this review, we summarize and discuss recent findings on the role of miRNAs in GCSC regulation. In addition, we perform a meta-analysis aimed to identify novel miRNAs involved in GCSC homeostasis.

## 1. Introduction

Gastric cancer (GC) is one of the most common malignant tumors and is associated with multiple genetic mutations and environmental interactions [1,2]. While in recent years several combinations of chemotherapy regimens have been tried, the improvement in survival rates is modest [3]. Indeed, GC still remains the third most common cause of cancer-related mortality [4,5]. 

As demonstrated by several studies, GC is characterized by a considerable heterogeneity at molecular, histological, and phenotypic levels [6], which plays a pivotal role in therapy resistance thus tumor recurrence [7,8].

The cancer stem cell (CSC) model of tumor progression theorizes that a small subpopulation of self-renewing cancer cells within tumor can sustain neoplasm growth and metastases spread [9,10], as well as cancer relapse and resistance to chemotherapy [11]. In CSCs, many of the normal stem cells properties, including self-renewal, differentiation, and proliferative potential, are dysregulated, principally due to epigenetic changes and genetic mutations [12].

The first evidence for a role of stem cells in cancer arose from the work of Lapidot et al., which reported that a small subpopulation of cells was able to reproduce leukemic disease in immunodeficient mice [13]. Since this finding, more and more studies have suggested the existence of CSCs in numerous solid tumors [14,15,16,17,18], including GC [19,20].

One of the most difficult challenges to target CSCs is to identify specific markers and, at the same time, to uncover targetable molecular features associated with their phenotype. To date, the molecular characterization of CSCs remains largely unknown.

Recent studies highlight the role of several microRNAs (miRNAs) as key regulators of molecular mechanisms that are associated with cancer drug resistance, making them an attractive therapeutic target [21]. According to recent theories that support the origin of cancer from CSCs, many studies suggest that miRNA dysregulation in CSCs may be involved in tumor growth and spread [22].

The miRNAs are categorized as a novel class of non-coding, single-strand, small RNAs. Based on their length, non-coding RNAs are classified in two groups: those with less than 200 nucleotides (smallRNAs), and those with more than 200 nucleotides, e.g., long non-coding RNAs (lncRNAs) and long intergenic non-coding RNAs (lincRNAs) [22]. The miRNAs are 21–25 nucleotides long, acting as post-transcriptional regulators through their binding at the untranslated region (mainly 3’-UTR (untranslated region)), of specific messenger RNAs (mRNAs), targeting their degradation or translation inhibition [23,24,25]. The first two miRNAs, lin-4 and let-7, were discovered in *Caenorhabditis elegans* [26]. Since their discovery, miRNAs were shown to play a specific role in the regulation of embryogenesis, stem/progenitor cells and CSCs biology [27]. Here, we review recent reports, indicating the key role of miRNAs in regulating CSCs, with a specific focus on gastric cancer stem cells (GCSCs), and we also report the results of a meta-analysis aimed at predicting a novel miRNA signature starting from GCSC global gene expression profiles.

## 2. MicroRNAs and Cancer Stem Cells

In this section we will discuss recent reports about relationship between miRNAs and cancer stem cells in different types of tumors (summarized in Table 1). 

In 2003 CSCs from breast cancer (BCSCs) were identified [13], and in 2007 Yu and colleagues identified let-7 which, by targeting *RAS* and *HMGA2* genes, is a master regulator for breast CSC properties, including self-renewal and multipotent differentiation capabilities [28]. Since then, several studies have confirmed the regulative role of miRNAs in the stem-like properties of BCSCs [29,30,31,32,33].

With respect to hematological malignancies, several abnormally regulated miRNAs were identified which target genes implicated in self-renewal, transformation, proliferation, and tumorigenicity [34,35,36,37]. Of note, miR-22 and miR-99, are oncogenic miRNAs which promote stem cell self-renewal [34,35]. Recently, Lechman et al. showed that miR-126 targeting the *PI3K/AKT/mTOR* pathway controls the cell cycle progression of leukemia stem cells (LSC) [36].

Numerous studies have also highlighted the important role of miRNAs in determining glioblastoma stem-like cells (GSCs) biological features [38,39,40,41]. In particular, miR-34a directly inhibits *c-Met* and *Notch-1/2* in glioma cells and stem cells through direct 3′-UTR binding [42]. Other miRNAs involved in the regulation of glioma cells stemness are: miR-125b and miR-29b [43,44].

In 2007, Ma et al. identified and isolated, for the first time, CSCs in liver cancer (LCSCs) [45]. To date, several miRNAs were reported to modulate self-renewal, proliferation, apoptosis, migration, invasion, and differentiation in LCSCs [46,47]. In particular, many studies underline the role of the let-7 family, miR-217 and miR-452 in the Wnt signaling pathway [48,49,50,51].

The miR-200 family, miR-203, miR-137, miR-34a, and miR-221, targeting various genes involved in the regulation of CSC properties, are considered to be the regulators of stem cell properties in colorectal CSCs [52]. A recent study reported that miR-508 is negatively correlated with the stem-like/mesenchymal colorectal cancer (CRC) subtype [53].

Fang et al. in 2015 identified, in prostate cancer stem cells (PCSCs), different miRNAs involved in the regulation of specific stemness-related surface markers and transcription factors [54]. Prostate tumor growth and metastasis formation capability are suppressed in PCSCs by miR-141, which targets genes such as *CD44*, *EZH2*, and *Rho GTPases* [55].

## 3. Gastric Cancer Stem Cells

There are two main hypotheses about the origin of GCSCs: the first one suggests that GCSCs derived from mutations of gastric stem cells (GSCs) which lead to sequential transformation of normal gastric mucosa to atrophic gastritis, intestinal metaplasia, atypical hyperplasia, and finally to GC [58]. Based on this hypothesis, it is crucial to evaluate the phenotypes of GCSCs in distinct anatomical regions and expressing different specific markers. In particular, the Lgr5^+^ subpopulation resides at the base of the pyloric glands, Villin^+^ cells are located at the bottom of antropyloric glands, Troy-expressing cells are located at the base of the corpus gastric glands, and Mist1^+^ and Sox2^+^ cells reside at the base of both corpus–fundus and antrum–pylorus regions and in the isthmus [59].

The second hypothesis is based on recent studies suggesting that bone marrow-derived cells (BMDCs) are candidates for GCSC [58]. In particular, Houghton et al. reported that, in mice with persistent *Helicobacter* infection, BMDCs migrate to the gastric mucosa and undergo malignant transformation into cancerous epithelial cells [60]. In 2012, Varon et al. confirmed that long-term *Helicobacter pylori* infection induces the recruitment and accumulation of BMDCs in the gastric epithelial mucosa, participating in GC progression [61]. In 2016, Zhang and colleagues supported this hypothesis [62].

CSCs can be isolated through fluorescence-activated cell sorting (FACS) and magnetic cell sorting (MACS), exploiting the presence of CSCs specific markers, as well as stem cell side population (SP) analysis [63,64,65]. About the different methods typically used to characterize CSCs, two phenotypic assays proved to be the most exhaustive. One is the in vitro “spheroid colony formation”, and the second is in vivo “tumorigenicity capability” through mouse model xenotransplantation [66,67]. In Table 2, we have listed the major surface markers or their combinations currently used to identify GCSCs able to generate both in vitro spheroid colonies and in vivo tumors.

## 4. MicroRNAs and Gastric Cancer Stem Cells

The expression of miRNAs has a pivotal role in the maintenance of stem/progenitor cells. Its perturbation is causative of the altered balance between self-renewal and differentiation that may cause a tumorigenic cellular phenotype [79]. Despite the considerable advances made in understanding the role of miRNAs in regulating GCSC biology, the mechanisms of action and the clinical utility of these regulatory RNA molecules are still far from being fully elucidated.

In 2017, Zeng et al. demonstrated, for the first time, that miR-145 inhibits the stem-like properties of GC targeting directly CD44 observing, at the same time, that the overexpression of miR-145 in GC was correlated with chemoresistance [80]. Furthermore, the miR-711 downregulated the CD44 expression causing the inhibition of epithelial to mesenchymal transition (EMT) in GC cells both in vitro and in vivo [81]. Moreover, in a recent study, Lee and colleagues underlined a relationship between the upregulation of miR-193a-3p and cisplatin resistance in CD44^+^ GC cells [82]. Functional studies have also shown that CD44^+^ cells exhibit a much more pronounced sphere-forming activity than CD44^-^ cells. A miRNA microarray analysis displayed that miR-196a-5p was upregulated in CD44^+^ cells and its suppression led to decreased colony formation and invasion of GCSCs suggesting a significant role of miR-196a-5p in EMT and invasion by targeting Smad4 in GCSCs [83]. Furthermore, in a recent study, Shao et al. reported that overexpression of lenti-miRNA-19b/20a/92a significantly enhanced the ability of GCSCs in forming tumor spheres [84].

Several signaling pathways shown to cooperatively ensure stem cell homeostasis are finely regulated. Abnormalities in their regulation may be responsible for the self-renewal unbalance of GCSCs [85]. However, it is well known that several specific miRNAs target genes related to key signal pathways involved in stemness regulation, such as Notch, Wnt/β-catenin, transforming growth factor-beta (TGF-β)/Smad, and Hippo signaling pathways.

Precisely, an investigation conducted by Xiao et al., demonstrated that miR-124 targeting *JAG1* suppress the Notch signaling pathway inhibiting invasion, migration, and proliferation of GC cells. [86].

Wu et al reported that miR-17-92, activates Wnt/β-catenin signaling, by targeting *E2F1* and *HIPK1* [87]. Another study suggested that miR-501-5p constitutively activates Wnt/β-catenin signaling by targeting *DKK1*, *NKD1*, and *GSK3β*, promoting a GCS-like phenotype [88]. According to these studies, a recent study proposed miRNA-194 as oncogene that promotes GC cell proliferation and migration by activating Wnt signaling and acting on the negative Wnt regulator SUFU (suppressor of fused homolog) [89]. 

In addition, Shao et al. in 2018, affirmed that miRNA-19b/20a/92a promotes GCSC self-renewal by targeting *E2F1* and *HIPK1* and activating the β-catenin signaling pathway [84]. 

In the same year, Song et al. proposed miR-338 as a putative tumor suppressor in GC which targeting *EphA2* blocks EMT leading to inhibition of Wnt/β-catenin signaling [90].

In 2014 Yu et al. displayed that miR-106b family expression modulated cancer stem-like cell properties, in particular EMT, via TGF-β/Smad signaling pathway in CD44^+^ stem-like cells [91]. Recent studies support the role of miRNAs in the regulation of Hippo pathway. First Li et al. reported that miR-93-5p promotes GC-cell progression through the inactivation of the Hippo signaling pathway [92]. Later in 2018, Kang and colleagues demonstrated the involvement of miR-375 in this pathway by targeting *YAP1/TEAD4-CTGF* axis in gastric tumorigenesis [93].

## 5. Meta-Analysis of Up/Down miRNAs in GCSCs Features: A Focus on Stemness-Related Pathways

In this section, we illustrate the results of a meta-analysis performed by using public microarray datasets to obtain a novel miRNA signature from genes resulted differentially expressed between GC cells and GCSCs. In particular, following PRISMA (preferred reporting items for systematic reviews and meta-analyses) guidelines (Figure S1), we selected data from two public datasets: GSE112631 (Illumina) and GSE20058 (Affymetrix). The first one, consisting of 12 samples, refers to gene expression data of CD133^+^ and CD133^−^ cells sorted from three GC cell lines (KATO-III, SNU216, and SNU601) [94].

The second dataset, consisting of four samples (resulting from a quality check on the original eight samples), characterizes the expression of stem cell side population (SP) of AGS cell line [95]. Briefly, after normalization performed accordingly to gene expression technologies (neqc and RMA), we used linear models and the empirical Bayes statistics by limma package [96] to assess differential gene expression data.

To perform meta-analysis, we combined *p*-values by Fisher’s sum of logs method by the metap package, followed by FDR multiple testing correction. Two significant (adjusted *p*-value < 0.05) differentially expressed genes (DEGs) lists were produced: a list of genes significantly highly expressed in GCSCs and a list of genes significantly highly expressed in GC cells. An enrichment analysis carried out using the clusterProfiler package [97], focused on miRNA targets (MSigDB collection [98,99]), revealed the miRNA were bona fide differentially expressed between the two conditions. We obtained eight (adjusted *p*-value < 0.05) predicted downregulated miRNAs (Table 3) from DEGs highly expressed in GCSCs and four (*p*-value < 0.05) predicted upregulated miRNAs (Table 4).

Interestingly, among the predicted miRNAs, which we hypothesized to have a key role in the GCSCs biology, we found several miRNAs previously identified [79,111]. 

In particular, in 2014 Liu et al. evaluated the miRNA expression profiles in spheroid body-forming cells and parental cells (MKN-45), finding182 miRNAs differentially expressed.

In conformity with the data obtained by Liu and colleagues, with respect to the downregulated predicted miRNAs we recovered miR-193A, miR-193B, and miR-93 [111].

Later, in 2017 Golestanech et al, studied the differentially expressed miRNAs in CSCs and cancer cells of the GC cell line MKN-45. According to their data, we identified, in the predicted miRNAs, an increase in miR-302 expression level and a downregulation of miR-372, miR-373 and miR-520C-5p in CSCs compared to in cancer cells [79].

Subsequently, we extended the enrichment analysis by DIANA-mirPath 3.0 [113] web server. Briefly, for each miRNA (both 5′ and 3′ arm), the algorithm retrieves the putative regulated genes and uses them to calculate the activation of each KEGG (kyoto encyclopedia of genes and genomes) pathway (Tables S1 and S2).

**Figure 1:**
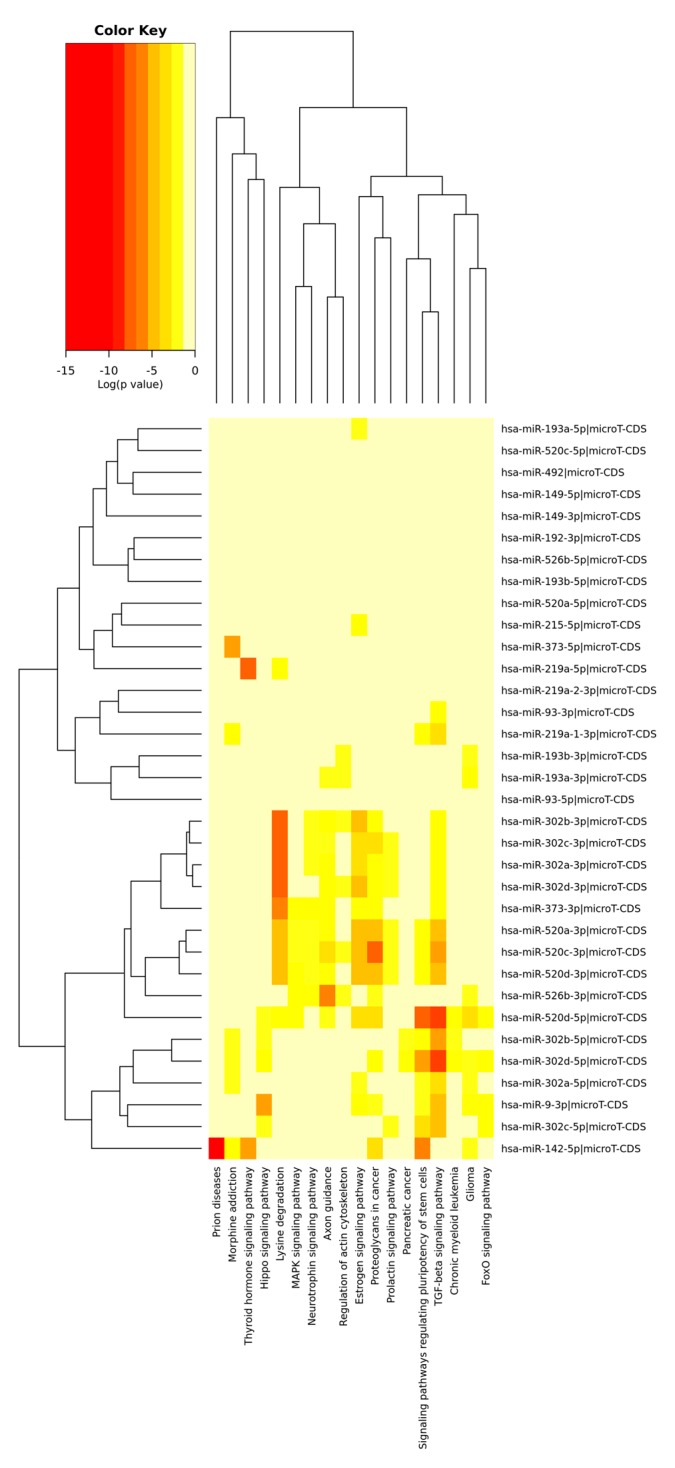
Heatmap of significant predicted downregulated miRNAs (both 5’ and 3’ arm) and relative KEGG activated pathways. The color in the heatmap represents the significance levels (*p*-values) between each miRNA and every pathway. A merged *p*-value is extracted by combining the previously calculated significance levels, using Fisher’s meta-analysis method. Thus, the resulting merged *p*-values signify if a particular pathway is targeted by at least one miRNA out of the initially selected group. T-CDS: microRNA target coding sequences; TGF: transforming growth factor.

**Figure 2:**
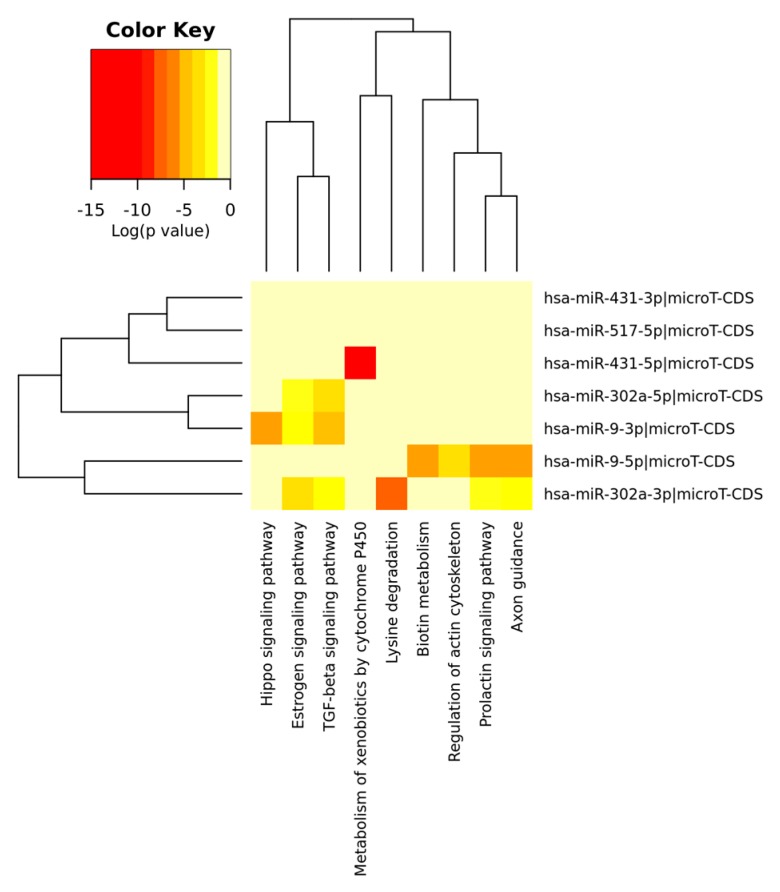
Heatmap of significant predicted upregulated miRNAs (both 5’ and 3’ arm) and relative KEGG activated pathways. The color in the heatmap represents the significance levels (*p*-values) between each miRNA and every pathway. A merged *p*-value is extracted by combining the previously calculated significance levels, using Fisher’s meta-analysis method. Thus, the resulting merged *p*-values signify if a particular pathway is targeted by at least one miRNA out of the initially selected group.

The Figure 1 and Figure 2 depict the heat maps of significant miRNAs and the relative KEGG activated pathways. All the analyses were performed by R/Bioconductor [114,115].

We focused our attention on the enrichment of the two stemness-related pathways, whose involvement in the genesis of GCSCs has long been established: Transforming growth factor-β (TGF-β) and Hippo signaling pathways. 

*TGF-β* has emerged as key regulator of stem cell self-renewal and differentiation [116]. A recent study suggests the putative oncogenic function of TGF-β in GC considering that its overexpression in the extracellular matrix (ECM) induces tumorigenicity [117]. Other evidence underlines that *H. pylori* infection might promote the TGF-β1-induced EMT in gastric mucosa and the development of GCSCs [118]. Moreover, bone marrow-derived mesenchymal stem cells (BM-MSCs) were shown to provide advantageous microenvironments for cancer progression by supporting proliferation, cluster formation, expansion of the CD133^+^ population, upregulation of *TGFβ1* and *WNT5A* genes in co-cultured MKN7 GC cells [119]. Furthermore, BM-MSCs promote GCSCs phenotype via TGF-β signaling in response to gastritis [120]. Peng et al. confirmed the role of *TGF-β/SOX4* axis in GC cells EMT and in the stemness regulation [121].

The Hippo signaling pathway is known as a tumor-suppressing pathway that acts on tissue homeostasis and organ size by inhibiting cell growth, proliferation and promoting apoptosis. The deregulation of Hippo signaling pathway is associated with initiation, development and metastasis spreading also in GC [122]. Moreover, Qiao et al. propose the Hippo pathway as a potential therapeutic target for GC treatment [123].

**Figure 3:**
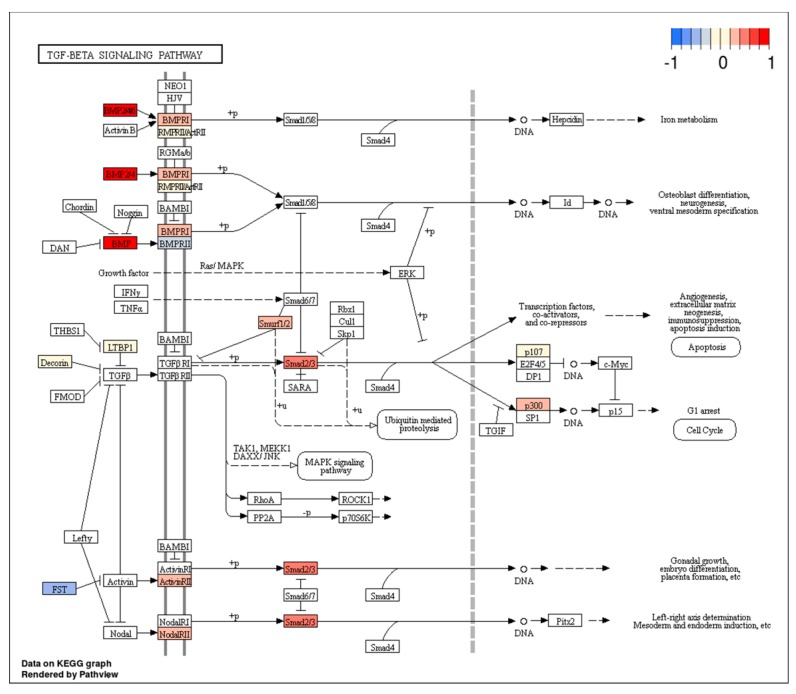
The transforming growth factor-β (TGF-β) signaling pathway with marked expression levels of deregulated genes. Arbitrary signal intensity acquired from microarray analysis is represented by colors (red, higher; blue, lower expression).

**Figure 4:**
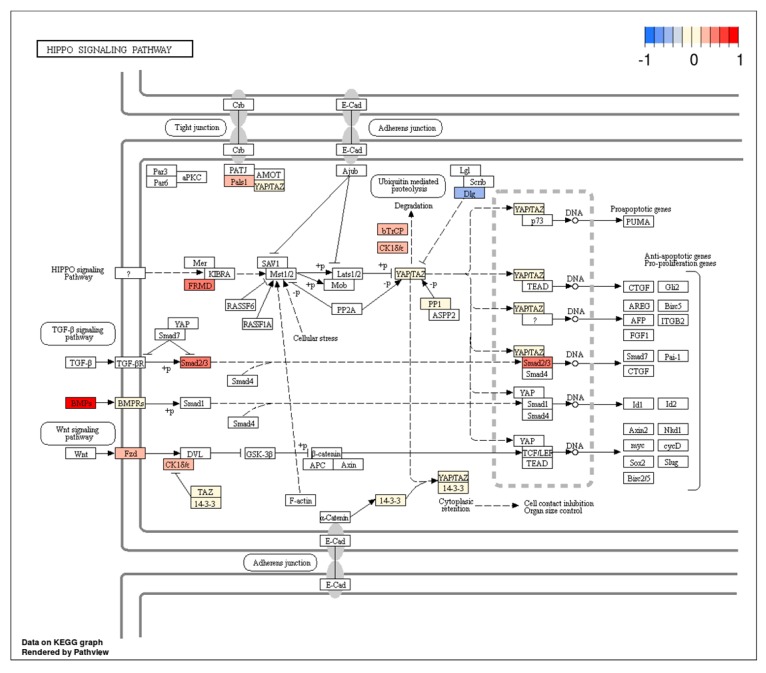
Hippo Signaling pathway with marked expression levels of deregulated genes. Arbitrary signal intensity acquired from microarray analysis is represented by colors (red, higher; blue, lower expression).

Figure 3 and Figure 4, processed by the pathview package [124], indicate several deregulated genes involved in these pathways. Their up or down expression may be related to specific miRNAs, which may represent potential GCSC targets.

With respect to the TGF-β pathway, the down expression of *Follistatin* (*FST*) seems interesting.

*FST* is an antagonist of the TGF-β superfamily member activin, a pleiotropic growth factor involved in proliferation, cancer progression, and cell invasion [125]. The roles of *FST* in tumorigenesis, progression, metastasis, and angiogenesis processes in different types of solid tumors [126] are known, and therefore we may suppose that the downregulation of FST, mediated by several miRNAs, could enhance the effects of activin and promote GCSC proliferation.

New in-depth studies, in this regard, could be useful to better understand the putative role of several miRNAs as regulators of TGF-β superfamily members’ antagonists and to possibly validate our hypothesis.

Among the up expressed genes involved in the Hippo signaling pathway, we hypothesize protein phosphatase 1 (PP1) to have a prominent role, as outlined below. The overexpression of *PP1* leads to dephosphorylation of *TAZ* and *YAP*. Unphosphorylated *TAZ* and *YAP* remain in the nucleus where they interact with *TEA/ATTS* domain (TEAD) transcription factors and promote cell proliferation, stem cell self-renewal, and tumorigenesis [127]. In line with our hypothesis, *YAP* and *TAZ* represent primary effectors of the Hippo pathway and have been recognized as important drivers of cancer progression and metastasis [128].

## 6. Conclusions

Overall, here we highlight the key role played by miRNAs in GCSC properties. Furthermore, our results indicate that other putative miRNAs partially ignored up to now should be studied. A better knowledge of miRNA molecular mechanisms and the gene targets involved in the regulation of GCSCs could open up new strategies in the target therapy of GCSCs.

However, due to the scarce availability of GCSCs global gene expression profile datasets, and the difficulties in retrieving them from open access repository miRNA GCSC datasets, the current meta-analysis is far from exhaustive. Therefore, new studies comprising integrated genes and miRNA expression data are strongly suggested.

## Figures and Tables

**Table 1:** Function of up/downregulated microRNA (miRNAs) in different types of cancer stem cells and their molecular targets.

MiRNA(s)	Target Gene(s)	Function	Cancer Type	Up/Downregulated	Reference(s)
**Let-7**	*RAS*, *HMGA2*	Regulation of self-renewal and multipotent differentiation capabilities	Breast	Down-regulated	Yu et al. [28]
**MiR-200 family**	*BMI1*	Regulator of stem cell self-renewal	Breast	Down-regulated	Shimono et al. [29]
	*Suz12*	Inhibition of mammosphere, in combination with chemotherapy suppression of tumor growth	Breast	Down-regulated	Iliopoulos and Lindahl-Allen [30]
	*ZEB1*, *ZEB2*, *TWIST1*	Transition to a breast cancer stem cell-like state	Breast	Down-regulated	Lim et al. [31]
**MiR-30 family**	*Ubc9*, *ITGB3*, *AVEN*	Involvement in to apoptosis, proliferation and in tumor initiating BCSCs	Breast	Down-regulated	Yu et al. [32] Ouzounova et al. [33]
**MiR-22**	*TET2*	Promotion of self- renewal and transformation	Hematological malignancies	Up-regulated	Song et al. [34]
**MiR-99**	*Hoxa1*	Regulation of self-renewal and multipotent differentiation capabilities	Hematological malignancies	Up-regulated	Khalaj et al. [35]
**MiR-126**	*PI3K/AKT/mTOR* pathway	Controller of cell cycle progression	Hematological malignancies	Up-regulated	Lechman et al. [36]
**MiR-150**	*Nanog*	Suppression of proliferation and tumorigenicity of LSC	Hematological malignancies	Down-regulated	Xu et al. [37]
**MiR-128**	*Bmi-1*	Regulator of stem cell self-renewal	Glioblastoma	Down-regulated	Godlewski et al. [39]
**MiR-451**	*PI3K/AKT*	Regulation of cell proliferation, invasion and apoptosis	Glioblastoma	Down-regulated	Gal et al. [40] Nan et al. [41]
**MiR-34a**	*c-Met* and *Notch-1/2*	Tumor suppressor	Glioblastoma	Down-regulated	Guessous et al. [42]
**MiR-125b**	*E2F2*	Regulation of cancer stem cell self-renewal and differentiation	Glioblastoma	Down-regulated	Wu et al. [43]
**MiR-29b**	*BCL2L2*	Attenuates tumorigenicity and stemness maintenance	Glioblastoma	Down-regulated	Chung et al [44].
**MiR-200a**	*ZEB2*	Inhibition of EMT	Liver	Down-regulated	Wang et al [46]
**MiR-491**	*GIT-1/NF-kb*	Inhibition of EMT	Liver	Down-regulated	Yang et al. [56]
**let-7 b**	*Frizzled 4*	Reduces the proportion of cancer stem cells	Liver	Down-regulated	Cai et al. [48]
**let-7 a**	*Wnt* pathway	Inhibition of EMT	Liver	Down-regulated	Jin et al. [49]
**MiR-217**	*DKK1*	Regulation of the CSC-like phenotypes	Liver	Up-regulated	Jiang et al. [50]
**MiR-452**	*SOX 7*	Promotion of stem-like cells	Liver	Down-regulated	Zheng et al. [51]
**MiR-200b/c, MiR-203**	*BMI1*, *ZEB1*	Coordinately function for the suppression of the stem cell properties of CSCs	Colorectal	Down-regulated	Mukohyama et al. [52]
**MiR-34a**	*Notch*	Maintenance of CSCs	Colorectal	Down-regulated	Mukohyama et al. [52]
**MiR-137**	*MSI1 DCLK1 FMNL1 CDC42*	Enhances the stem cell properties of CSCs	Colorectal	Up-regulated	Mukohyama et al. [52]
**MiR-221**	*PTEN p27*, *p57*				
**MiR-508**	*ZEB1*, *BMI1*, and *SALL4*,	Inhibits the CRC EMT and stemness process.	Colorectal	Down-regulated	Yan et al. [53]
**MiR-320**	*Wnt/*beta-catenin signaling pathway	Promotes cancer stem cell-like properties	Prostate	Down-regulated	Hsieh et al. [57]
**MiR-141**	*CD44*, *EZH2* and *Rho GTPases*	Suppresses tumor growth and metastasis	Prostate	Down-regulated	Liu et al. [55]

CSCs: cancer stem cells; BCSCs: CSCs from breast cancer; LSC: leukemia stem cells; CRC: colorectal cancer; EMT: epithelial to mesenchymal transition; MiR: microRNA.

**Table 2:** Gastric cancer markers used to characterize gastric cancer stem cells.

Marker(s) Expression	In Vitro Assay	Efficiency (%) ^a^	In Vivo Assay	Efficiency (%) ^b^	Reference(s)
**CD44^+^**	Spheroid colony formation	10 cells/well NCI-N87, MKN-74, MKN-45 ^1^ 20 cells/well Human gastric cancer tissues ^2^	Tumorigenicity (SCID mice) ^1^ (Nude mice) ^2^	20,000–30,000 cells injected ^1^ Skin NCI-N87, MKN-74, MKN-45 (100% efficiency) Stomach MKN-45 (100% efficiency) MKN-74 (75% efficiency) NCI-N87 (50% efficiency) 10,000 cells injected ^2^ Human gastric cancer tissues (80% efficiency)	1. Takaishi et al. [20] 2. Sun et al. [68]
**CD44^+^/CD24^+^**	Tumoroid sphere formation	100 cell/well AGS	Tumorigenicity (NOD/SCID mice)	200 cells injected AGS (50% efficiency)	Zhang et al. [69]
**CD44^+^/CD54^+^**	Spheroid formation	1 cell/well Human gastric cancer tissues 10,000 cells/dish Blood samples	Tumorigenicity (SCID/Nude mice)	1000 cells injected Human gastric cancer tissues (100% efficiency) 9000 cells from spheres injected gastric cancer cells in circulation	Chen et al. [70]
**CD44^+^/CD26^+^**	Spheroid formation	≤5 × 10^6^ cells/dish MKN7, MKN28, MKN45, AZ521	Tumorigenicity (NOD/SCID mice)	100 cells injected Human gastric cancer tissues (100% efficiency)	Nishikawa et al. [71]
**CD44^+^/EpCAM^+^**	Spheroid formation	1 cell/well Human gastric cancer tissues	Tumorigenicity (Nude mice)	500 cells injected Human gastric cancer tissues (50% efficiency)	Han et al. [72]
**CD44v8–10+**	Spheroid formation	100 cells/dish (35-mm) GC45, GC84 xenograft tumors	Tumorigenicity (NOD/SCID mice)	200 cells injected (75% efficiency)	Lau et al. [73]
**CD71^−^**	Colony formation	500–1000 cells/dish (35-mm) MKN-1	Tumorigenicity (NOD/SCID mice)	100 cells injected (80% efficiency)	Ohkuma M. et al. [74]
**CD90^+^**	Spheroid formation	5000 cells/mL Gastric primary tumor model	Tumorigenicity (Nude mice)	100 cells injected High Tumorigenicity group (100% efficiency)	Jiang et al. [75]
**CD133^+^**	Colony formation	1 cell/well KATO-III	Tumorigenicity (Nude mice)	10,000 cells injected (100% efficiency)	Chen et al. [76]
**ALDH1^+^**	Colony formation	20,000 cells/well OCUM2-LMN	Tumorigenicity (Nude mice)	100 cells injected (100% efficiency)	Katsuno et al. [77]
**LGR5^+^**	Spheroid formation	10,000 cells/well MKN-45, MKN-28	Tumorigenicity (Nude mice)	10,000 cells injected MKN-45, MKN-28 (100% efficiency)	Zhang et al. [78]

The most representative gastric cancer markers used to characterize gastric cancer stem cells. The markers listed have the ability to generate spheroid colony and demonstrable tumorigenicity capability. Efficiency was expressed as: (a) the minimum number of cells to generate spheroid colony; and (b) the minimum number of sorted cells injected to ensure at last 50% of tumorigenicity. SCID: severe combined immunodeficient mice; Nude mice: Balb/cA nu/nu female mice; NOD/SCID: non/obese diabetic/severe combined immunodeficient.

**Table 3:** List of downregulated miRNAs in gastric cancer stem cell.  Adjusted *p*-value <  0.05 was considered as statistically significant.

ID	Motif	Adjusted *p*-Value	Functional Relevance in GC
MiR-93	AGCACTT	0.017	OncomiR [92]
MiR-302A_MiR-302B_MiR-302C			Tumor suppressor [100,101,102]
MiR-302D			No data
MiR-372_MiR-373_ MiR-520C			Tumor suppressor [79]
MiR-520E_MiR-520A_ MiR-520B _MiR-520D			Tumor suppressor [103]
MiR-526B			Tumor suppressor [104]
MiR-149	GAGCCAG	0.030	Tumor suppressor [105]
MiR-9	ACCAAAG	0.030	Tumor suppressor [106]
MiR-219	GACAATC	0.031	Tumor suppressor [107]
MiR-193A_MiR-193B	GGCCAGT	0.031	OncomiR [108]
MiR-492	CAGGTCC	0.031	No data
MiR-142_5P	ACTTTAT	0.031	Tumor suppressor [109]
MiR-192_MiR-215	TAGGTCA	0.039	Tumor suppressor [110]

If not indicated, the motif could target the 5’ arm, the 3’ arm or both. GC: Gastric cancer; MiR: microRNA; OncomiR: oncogenic microRNA.

**Table 4:** List of upregulated miRNAs in gastric cancer stem cells. *p*-value < 0.05 was considered as statistically significant.

ID	Motif	*p*-Value	Functional Relevance in GC
MiR-9	TAGCTTT	0.007	OncomiR [112]
MiR-431	GCAAGAC	0.014	No data
MiR-302A	CACGTTT	0.025	OncomiR [79]
MiR-517	TCTAGAG	0.040	No data

Motif could target the 5’ arm, the 3’ arm or both. GC: gastric cancer; MiR: microRNA; OncomiR: oncogenic microRNA.

## Supplementary Materials

The following are available online at https://www.mdpi.com/2077-0383/8/5/639/s1. Table S1: Significant KEGG pathways by predicted upregulated miRNAs; Table S2: Significant KEGG pathways by predicted downregulated miRNAs; Figure S1: PRISMA flow diagram.

## References

[1] McLean M.H., El-Omar E.M. (2014). Genetics of gastric cancer. Nat. Rev. Gastroenterol. Hepatol..

[2] Siegel R.L., Miller K.D., Jemal A. (2016). Cancer statistics, 2016. CA Cancer J. Clin..

[3] Bernards N., Creemers G.J., Nieuwenhuijzen G.A.P., Bosscha K., Pruijt J.F.M., Lemmens V.E.P.P. (2013). No improvement in median survival for patients with metastatic gastric cancer despite increased use of chemotherapy. Ann. Oncol..

[4] Ferlay J., Soerjomataram I., Dikshit R., Eser S., Mathers C., Rebelo M., Parkin D.M., Forman D., Bray F. (2015). Cancer incidence and mortality worldwide: Sources, methods and major patterns in GLOBOCAN 2012. Int. J. Cancer.

[5] Torre L.A., Bray F., Siegel R.L., Ferlay J., Lortet-Tieulent J., Jemal A. (2015). Global cancer statistics, 2012. CA Cancer J. Clin..

[6] Gullo I., Carneiro F., Oliveira C., Almeida G.M. (2018). Heterogeneity in Gastric Cancer: From Pure Morphology to Molecular Classifications. Pathobiology.

[7] Gao J.P., Xu W., Liu W.T., Yan M., Zhu Z.G. (2018). Tumor heterogeneity of gastric cancer: From the perspective of tumor-initiating cell. World J. Gastroenterol..

[8] McGranahan N., Swanton C. (2017). Clonal Heterogeneity and Tumor Evolution: Past, Present, and the Future. Cell.

[9] Baccelli I., Trumpp A. (2012). The evolving concept of cancer and metastasis stem cells. J. Cell Biol..

[10] Kreso A., Dick J.E. (2014). Evolution of the cancer stem cell model. Cell Stem Cell.

[11] Iseghohi S.O. (2016). Cancer stem cells may contribute to the difficulty in treating cancer. Genes Dis..

[12] Clarke M.F., Dick J.E., Dirks P.B., Eaves C.J., Jamieson C.H.M., Jones D.L., Visvader J., Weissman I.L., Wahl G.M. (2006). Cancer stem cells-perspective on current status and future directions: AACR workshop on cancer stem cells. Cancer Res..

[13] Lapidot T., Sirard C., Vormoor J., Murdoch B., Hoang T., Caceres-Cortes J., Minden M., Paterson B., Caligiuri M.A., Dick J.E. (1994). A cell initiating human acute myeloid leukaemia after transplantation into SCID mice. Nature.

[14] Al-Hajj M., Wicha M.S., Benito-Hernandez A., Morrison S.J., Clarke M.F. (2003). Prospective identification of tumorigenic breast cancer cells. Proc. Natl. Acad. Sci. USA.

[15] Singh S.K., Clarke I.D., Terasaki M., Bonn V.E., Hawkins C., Squire J., Dirks P.B. (2003). Identification of a cancer stem cell in human brain tumors. Cancer Res..

[16] Collins A.T., Berry P.A., Hyde C., Stower M.J., Maitland N.J. (2005). Prospective identification of tumorigenic prostate cancer stem cells. Cancer Res..

[17] Fang D., Nguyen T.K., Leishear K., Finko R., Kulp A.N., Hotz S., Van Belle P.A., Xu X., Elder D.E., Herlyn M. (2005). A tumorigenic subpopulation with stem cell properties in melanomas. Cancer Res..

[18] Li C., Heidt D.G., Dalerba P., Burant C.F., Zhang L., Adsay V., Wicha M., Clarke M.F., Simeone D.M. (2007). Identification of pancreatic cancer stem cells. Cancer Res..

[19] Yashiro M. (2014). Gastric Cancer Stem Cells and Resistance to Cancer Therapy. Chemotherapy.

[20] Takaishi S., Okumura T., Tu S., Wang S.S., Shibata W., Vigneshwaran R., Gordon S.A., Shimada Y., Wang T.C. (2009). Identification of gastric cancer stem cells using the cell surface marker CD44. Stem Cells.

[21] Riquelme I., Letelier P., Riffo-Campos A.L., Brebi P., Roa J.C. (2016). Emerging Role of miRNAs in the Drug Resistance of Gastric Cancer. Int. J. Mol. Sci..

[22] Ali Hosseini Rad S., Bavarsad M.S., Arefian E., Jaseb K., Shahjahani M., Saki N. (2013). The Role of microRNAs in Stemness of Cancer Stem Cells. Oncol. Rev..

[23] Iorio M.V., Croce C.M. (2012). MicroRNA dysregulation in cancer: Diagnostics, monitoring and therapeutics. A comprehensive review. EMBO Mol. Med..

[24] Croce C.M., Calin G.A. (2005). miRNAs, cancer, and stem cell division. Cell.

[25] Farazi T.A., Hoell J.I., Morozov P., Tuschl T. (2013). MicroRNAs in human cancer. Adv. Exp. Med. Biol..

[26] Lee R.C., Feinbaum R.L., Ambros V. (1993). The *C. elegans* heterochronic gene lin-4 encodes small RNAs with antisense complementarity to lin-14. Cell.

[27] Pasquinelli A.E., Reinhart B.J., Slack F., Martindale M.Q., Kuroda M.I., Maller B., Hayward D.C., Ball E.E., Degnan B., Müller P. (2000). Conservation of the sequence and temporal expression of let-7 heterochronic regulatory RNA. Nature.

[28] Yu F., Yao H., Zhu P., Zhang X., Pan Q., Gong C., Huang Y., Hu X., Su F., Lieberman J., Song E. (2007). Let-7 regulates self-renewal and tumorigenicity of breast cancer cells. Cell.

[29] Shimono Y., Zabala M., Cho R.W., Lobo N., Dalerba P., Qian D., Diehn M., Liu H., Panula S.P., Chiao E. (2009). Downregulation of miRNA-200c links breast cancer stem cells with normal stem cells. Cell.

[30] Iliopoulos D., Lindahl-Allen M. (2010). Loss of miR-200 inhibition of Suz12 leads to polycomb-mediated repression required for the formation and maintenance of cancer stem cells. Mol. Cell.

[31] Lim Y., Wright J., Attema J., Gregory P., Bert A., Smith E., Thomas D., Drew P., Khew-Goodall Y., Goodall G. (2013). Epigenetic modulation of the miR-200 family is associated with transition to a breast cancer stem cell-like state. J. Cell Sci..

[32] Yu F., Deng H., Yao H., Liu Q., Su F., Song E. (2010). MiR-30 reduction maintains self-renewal and inhibits apoptosis in breast tumor-initiating cells. Oncogene.

[33] Ouzounova M., Vuong T., Ancey P.B., Ferrand M., Durand G., Le-Calvez Kelm F., Croce C., Matar C., Herceg Z., Hernandez-Vargas H. (2013). MicroRNA miR-30 family regulates non-attachment growth of breast cancer cells. BMC Genom..

[34] Song S.J., Ito K., Ala U., Kats L., Webster K., Sun S.M., Jongen-Lavrencic M., Manova-Todorova K., Teruya-Feldstein J., Avigan D.E. (2013). The oncogenic microRNA miR-22 targets the TET2 tumor suppressor to promote hematopoietic stem cell self-renewal and transformation. Cell Stem Cell.

[35] Khalaj M., Woolthuis C.M., Hu W., Durham B.H., Chu S.H., Qamar S., Armstrong S.A., Park C.Y. (2017). miR-99 regulates normal and malignant hematopoietic stem cell self-renewal. J. Exp. Med..

[36] Lechman E.R., Gentner B., Ng S.W., Schoof E.M., van Galen P., Kennedy J.A., Nucera S., Ciceri F., Kaufmann K.B., Takayama N. (2016). miR-126 Regulates Distinct Self-Renewal Outcomes in Normal and Malignant Hematopoietic Stem Cells. Cancer Cell.

[37] Xu D.D., Zhou P.J., Wang Y., Zhang Y., Zhang R., Zhang L., Chen S.H., Fu W.Y., Ruan B.B., Xu H.P. (2016). MiR-150 suppresses the proliferation and tumorigenicity of leukemia stem cells by targeting the nanog signaling pathway. Front. Pharm..

[38] Kleinová R., Slabý O., Šána J. (2015). Te Relevance of MicroRNAs in Glioblastoma Stem Cells. Klin. Onkol. Cas Ceské Slov. Onkol. Spol..

[39] Godlewski J., Nowicki M.O., Bronisz A., Williams S., Otsuki A., Nuovo G., Raychaudhury A., Newton H.B., Chiocca E.A., Lawler S. (2008). Targeting of the Bmi-1 oncogene/stem cell renewal factor by microRNA-128 inhibits glioma proliferation and self-renewal. Cancer Res..

[40] Gal H., Pandi G., Kanner A.A., Ram Z., Lithwick-Yanai G., Amariglio N., Rechavi G., Givol D. (2008). MIR-451 and Imatinib mesylate inhibit tumor growth of Glioblastoma stem cells. Biochem. Biophys. Res. Commun..

[41] Nan Y., Han L., Zhang A., Wang G., Jia Z., Yang Y., Yue X., Pu P., Zhong Y., Kang C. (2010). MiRNA-451 plays a role as tumor suppressor in human glioma cells. Brain Res..

[42] Guessous F., Zhang Y., Kofman A., Catania A., Li Y., Schiff D., Purow B., Abounader R. (2010). microRNA-34a is tumor suppressive in brain tumors and glioma stem cells. Cell Cycle.

[43] Wu N., Xiao L., Zhao X., Zhao J., Wang J., Wang F., Cao S., Lin X. (2012). miR-125b regulates the proliferation of glioblastoma stem cells by targeting E2F2. FEBS Lett..

[44] Chung H.J., Choi Y.E., Kim E.S., Han Y.H., Park M.J., Bae I.H. (2015). miR-29b attenuates tumorigenicity and stemness maintenance in human glioblastoma multiforme by directly targeting BCL2L2. Oncotarget.

[45] Ma S., Chan K.W., Hu L., Lee T.K., Wo J.Y., Ng I.O., Zheng B.J., Guan X.Y. (2007). Identification and characterization of tumorigenic liver cancer stem/progenitor cells. Gastroenterology.

[46] Wang J., Yang X., Ruan B., Dai B., Gao Y., Duan J., Qu S., Tao K., Dou K., Li H. (2015). Overexpression of miR-200a suppresses epithelial-mesenchymal transition of liver cancer stem cells. Tumour Biol..

[47] Lou W., Liu J., Gao Y., Zhong G., Ding B., Xu L., Fan W. (2018). MicroRNA regulation of liver cancer stem cells. Am. J. Cancer Res..

[48] Cai H., Chen Y., Yang X., Ma S., Wang Q., Zhang Y., Niu X., Ding G., Yuan Y. (2017). Let7b modulates the Wnt/beta-catenin pathway in liver cancer cells via downregulated Frizzled4. Tumour Biol..

[49] Jin B., Wang W., Meng X.X., Du G., Li J., Zhang S.Z., Zhou B.H., Fu Z.H. (2016). Let-7 inhibits self-renewal of hepatocellular cancer stem-like cells through regulating the epithelial-mesenchymal transition and the Wnt signaling pathway. BMC Cancer.

[50] Jiang C., Yu M., Xie X., Huang G., Peng Y., Ren D., Lin M., Liu B., Liu M., Wang W., Kuang M. (2017). miR-217 targeting DKK1 promotes cancer stem cell properties via activation of the Wnt signaling pathway in hepatocellular carcinoma. Oncol. Rep..

[51] Zheng Z., Liu J., Yang Z., Wu L., Xie H., Jiang C., Lin B., Chen T., Xing C., Liu Z. (2016). MicroRNA-452 promotes stem-like cells of hepatocellular carcinoma by inhibiting Sox7 involving Wnt/beta-catenin signaling pathway. Oncotarget.

[52] Mukohyama J., Shimono Y., Minami H., Kakeji Y., Suzuki A. (2017). Roles of microRNAs and RNA-Binding Proteins in the Regulation of Colorectal Cancer Stem Cells. Cancers.

[53] Yan T.T., Ren L.L., Shen C.Q., Wang Z.H., Yu Y.N., Liang Q., Tang J.Y., Chen Y.X., Sun D.F., Zgodziński W. (2018). MicroRNA-508 defines the stem-like/mesenchymal subtype in colorectal cancer. Cancer Res..

[54] Fang Y.X., Chang Y.L., Gao W.Q. (2015). MicroRNAs targeting prostate cancer stem cells. Exp. Biol. Med..

[55] Liu C., Liu R., Zhang D., Deng Q., Liu B., Chao H.P., Rycaj K., Takata Y., Lin K., Lu Y. (2017). MicroRNA-141 suppresses prostate cancer stem cells and metastasis by targeting a cohort of pro-metastasis genes. Nat. Commun..

[56] Yang X., Ye J., Yan H., Tang Z., Shen J., Zhang J., Yang L. (2016). MiR-491 attenuates cancer stem cells-like properties of hepatocellular carcinoma by inhibition of GIT-1/NF-kappaB-mediated EMT. Tumour Biol..

[57] Hsieh I.S., Chang K.C., Tsai Y.T., Ke J.Y., Lu P.J., Lee K.H., Yeh S.D., Hong T.M., Chen Y.L. (2013). MicroRNA-320 suppresses the stem cell-like characteristics of prostate cancer cells by downregulating the Wnt/beta-catenin signaling pathway. Carcinogenesis.

[58] Fu Y., Li H., Hao X. (2017). The self-renewal signaling pathways utilized by gastric cancer stem cells. Tumour Biol..

[59] Huang Q., Zou X. (2017). Clinicopathology of Early Gastric Carcinoma: An Update for Pathologists and Gastroenterologists. Gastrointest. Tumors.

[60] Houghton J., Stoicov C., Nomura S., Rogers A.B., Carlson J., Li H., Cai X., Fox J.G., Goldering J.R., Wang T.C. (2004). Gastric cancer originating from bone marrow-derived cells. Science.

[61] Varon C., Dubus P., Mazurier F., Asencio C., Chambonnier L., Ferrand J., Giese A., Senant-Dugot N., Carlotti M., Mégraud F. (2012). Helicobacter pylori infection recruits bone marrow-derived cells that participate in gastric preneoplasia in mice. Gastroenterology.

[62] Zhang S., Kim W., Pham T.T., Rogers A.B., Houghton J.M., Moss S.F. (2016). Native and bone marrow-derived cell mosaicism in gastric carcinoma in H. pylori-infected p27-deficient mice. Oncotarget.

[63] Telford W.G. (2010). Stem cell side population analysis and sorting using DyeCycle violet. Curr. Protoc. Cytom..

[64] Greve B., Kelsch R., Spaniol K., Eich H.T., Gotte M. (2012). Flow Cytometry in Cancer Stem Cell Analysis and Separation. Cytom. A.

[65] Shimoda M., Ota M., Okada Y. (2018). Isolation of cancer stem cells by side population method. Methods Mol. Biol..

[66] Pastrana E., Silva-Vargas V., Doetsch F. (2011). Eyes wide open: A critical review of sphere-formation as an assay for stem cells. Cell Stem Cell.

[67] Tang D.G. (2012). Understanding cancer stem cell heterogeneity and plasticity. Cell Res..

[68] Sun M., Zhou W., Zhang Y.Y., Wang D.L., Wu X.L. (2013). CD44+ gastric cancer cells with stemness properties are chemoradioresistant and highly invasive. Oncol. Lett..

[69] Zhang C., Li C., He F., Cai Y., Yang H. (2011). Identification of CD44+CD24+ gastric cancer stem cells. J. Cancer Res. Clin. Oncol..

[70] Chen T., Yang K., Yu J., Meng W., Yuan D., Bi F., Liu F., Liu J., Dai B., Chen X. (2012). Identification and expansion of cancer stem cells in tumor tissues and peripheral blood derived from gastric adenocarcinoma patients. Cell Res..

[71] Nishikawa S., Konno M., Hamabe A., Hasegawa S., Kano Y., Fukusumi T., Satoh T., Takiguchi S., Mori M., Doki Y. (2015). Surgically resected human tumors reveal the biological significance of the gastric cancer stem cell markers CD44 and CD26. Oncol. Lett..

[72] Han M.E., Jeon T.Y., Hwang S.H., Lee Y.S., Kim H.J., Shim H.E., Yoon S., Baek S.Y., Kim B.S., Kang C.D. (2011). Cancer spheres from gastric cancer patients provide an ideal model system for cancer stem cell research. Cell Mol. Life Sci..

[73] Lau W.M., Teng E., Chong H.S., Lopez K.A., Tay A.Y., Salto-Tellez M., Shabbir A., So J.B., Chan S.L. (2014). CD44v8-10 is a cancer-specific marker for gastric cancer stem cells. Cancer Res..

[74] Ohkuma M., Haraguchi N., Ishii H., Mimori K., Tanaka F., Kim H.M., Shimomura M., Hirose H., Yanaga K., Mori M. (2012). Absence of CD71 transferrin receptor characterizes human gastric adenosquamous carcinoma stem cells. Ann. Surg. Oncol..

[75] Jiang J., Zhang Y., Chuai S., Wang Z., Zheng D., Xu F., Zhang Y., Li C., Liang Y., Chen Z. (2012). Trastuzumab (Herceptin) targets gastric cancer stem cells characterized by CD90 phenotype. Oncogene.

[76] Chen D.H., Lu R.Q., Ni X.C., Wu J.G., Wang S.L., Jiang B.J., Yu J.W. (2015). Influence of CD133+ expression on patients’ survival and resistance of CD133+ cells to anti-tumor reagents in gastric cancer. Asian Pac. J. Trop. Biomed..

[77] Katsuno Y., Ehata S., Yashiro M., Yanagihara K., Hirakawa K., Miyazono K. (2012). Coordinated expression of REG4 and aldehyde dehydrogenase 1 regulating tumourigenic capacity of diffuse-type gastric carcinoma initiating cells is inhibited by TGF-β. J. Pathol..

[78] Zhang L., Guo X., Zhang D., Fan Y., Qin L., Dong S., Zhang L. (2017). Upregulated miR-132 in Lgr5+ gastric cancer stem cell-like cells contributes to cisplatin-resistance via SIRT1/CREB/ABCG2 signaling pathway. Mol. Carcinog..

[79] Golestaneh A.F., Atashi A., Langroudi L., Shafiee A., Ghaemi N., Soleimani M. (2012). miRNAs expressed differently in cancer stem cells and cancer cells of human gastric cancer cell line MKN-45. Cell Biochem. Funct..

[80] Zeng J.F., Ma X.Q., Wang L.P., Wang W. (2017). MicroRNA-145 exerts tumor-suppressive and chemo-resistance lowering effects by targeting CD44 in gastric cancer. World J. Gastroenterol..

[81] Xiao W., Li D., Tang Y., Chen Y., Deng W.B., Chen J., Zhou W.W., Liao A. (2018). Inhibition of epithelialmesenchymal transition in gastric cancer cells by miR-711-mediated downregulation of CD44 expression. Oncol. Rep..

[82] Lee S.D., Yu D., Lee D.Y., Shin H.S., Jo J.H., Lee Y.C. (2019). Upregulated miR-193a-3p is responsible for cisplatin resistance in CD44(+) gastric cancer cells. Cancer Sci..

[83] Pan Y., Shu X., Sun L., Yu L., Sun L., Yang Z., Ran Y. (2017). miR196a5p modulates gastric cancer stem cell characteristics by targeting Smad4. Int. J. Oncol..

[84] Shao Q., Xu J., Guan X., Zhou B., Wei W., Deng R., Li D., Xu X., Zhu H. (2018). In vitro and in vivo effects of miRNA-19b/20a/92a on gastric cancer stem cells and the related mechanism. Int. J. Med. Sci..

[85] Beachy P.A., Karhadkar S.S., Berman D.M. (2004). Tissue repair and stem cell renewal in carcinogenesis. Nature.

[86] Xiao H.J., Ji Q., Yang L., Li R.T., Zhang C., Hou J.M. (2017). In vivo and in vitro effects of microRNA-124 on human gastric cancer by targeting JAG1 through the Notch signaling pathway. J. Cell. Biochem..

[87] Wu Q., Yang Z., Wang F., Hu S., Yang L., Shi Y., Fan D. (2013). MiR-19b/20a/92a regulates the self-renewal and proliferation of gastric cancer stem cells. J. Cell Sci..

[88] Fan D., Ren B., Yang X., Liu J., Zhang Z. (2016). Upregulation of miR-501-5p activates the wnt/β-catenin signaling pathway and enhances stem cell-like phenotype in gastric cancer. J. Exp. Clin. Cancer Res..

[89] Peng Y., Zhang X., Ma Q., Yan R., Qin Y., Zhao Y., Cheng Y., Yang M., Wang Q., Feng X. (2017). MiRNA-194 activates the Wnt/β-catenin signaling pathway in gastric cancer by targeting the negative Wnt regulator, SUFU. Cancer Lett..

[90] Song B., Lin H.X., Dong L.L., Ma J.J., Jiang Z.G. (2018). MicroR- NA-338 inhibits proliferation, migration, and invasion of gastric cancer cells by the Wnt/β-catenin signaling pathway. Eur. Rev. Med. Pharm. Sci..

[91] Yu D., Shin H.S., Lee Y.S., Lee Y.C. (2014). miR-106b modulates cancer stem cell characteristics through TGF-beta/Smad signaling in CD44-positive gastric cancer cells. Lab. Investig..

[92] Li L., Zhao J., Huang S., Wang Y., Zhu L., Cao Y., Xiong J., Deng J. (2018). MiR-93-5p promotes gastric cancer-cell progression via inactivation of the Hippo signaling pathway. Gene.

[93] Kang W., Huang T., Zhou Y., Zhang J., Lung R.W.M., Tong J.H.M., Chan A.W.H., Zhang B., Wong C.C., Wu F. (2018). miR-375 is involved in Hippo pathway by targeting YAP1/TEAD4-CTGF axis in gastric carcinogenesis. Cell Death Dis..

[94] Kim T.M., Ko Y.H., Ha S.J., Lee H.H. (2019). Impact of in vitro driven expression signatures of CD133 stem cell marker and tumor stroma on clinical outcomes in gastric cancers. BMC Cancer.

[95] Schmuck R., Warneke V., Behrens H.M., Simon E., Weichert W., Röcken C. (2011). Genotypic and phenotypic characterization of side population of gastric cancer cell lines. Am. J. Pathol..

[96] Ritchie M.E., Phipson B., Wu D., Hu Y., Law C.W., Shi W., Smyth G.K. (2015). limma powers differential expression analyses for RNA-sequencing and microarray studies. Nucleic Acids Res..

[97] Yu G., Wang L.G., Han Y., He Q.Y. (2012). clusterProfiler: An R package for comparing biological themes among gene clusters. OMICS.

[98] Subramanian A., Tamayo P., Mootha V.K., Mukherjee S., Ebert B.L., Gillette M.A., Paulovich A., Pomeroy S.L., Golub T.R., Lander E.S. (2005). Gene set enrichment analysis: A knowledge-based approach for interpreting genome-wide expression profiles. Proc. Natl. Acad. Sci. USA.

[99] Liberzon A., Birger C., Thorvaldsdóttir H., Ghandi M., Mesirov J.P., Tamayo P. (2015). The Molecular Signatures Database (MSigDB) hallmark gene set collection. Cell Syst..

[100] Khalili M., Sadeghizadeh M., Ghorbanian K., Malekzadeh R., Vasei M., Mowla S.J. (2012). Down-regulation of miR-302b, an ESC-specific microRNA, in Gastric Adenocarcinoma. Cell J..

[101] Ma G., Li Q., Dai W., Yang X., Sang A. (2017). Prognostic Implications of miR-302a/b/c/d in Human Gastric Cancer. Pathol. Oncol. Res..

[102] Chen L., Min L., Wang X., Zhao J., Chen H., Qin J., Chen W., Shen Z., Tang Z., Gan Q., Ruan Y. (2015). Loss of RACK1 Promotes Metastasis of Gastric Cancer by Inducing a miR-302c/IL8 Signaling Loop. Cancer Res..

[103] Li S., Zhang H., Ning T., Wang X., Liu R., Yang H., Han Y., Deng T., Zhou L., Zhang L. (2016). MiR-520b/e Regulates Proliferation and Migration by Simultaneously Targeting EGFR in Gastric Cancer. Cell Physiol. Biochem..

[104] Fan Q.-H., Yu R., Huang W.-X., Cui X.-X., Luo B.-H., Zhang L.-Y. (2014). The has-miR-526b binding-site rs8506G>a polymorphism in the lincRNA-NR_024015 exon identified by GWASs predispose to non-cardia gastric cancer risk. PLoS ONE.

[105] Wang Y., Zheng X., Zhang Z., Zhou J., Zhao G., Yang J., Xia L., Wang R., Cai X., Hu H. (2012). MicroRNA-149 inhibits proliferation and cell cycle progression through the targeting of ZBTB2 in human gastric cancer. PLoS ONE.

[106] Zheng L., Qi T., Yang D., Qi M., Li D., Xiang X., Huang K., Tong Q. (2013). microRNA-9 suppresses the proliferation, invasion and metastasis of gastric cancer cells through targeting cyclin D1 and Ets1. PLoS ONE.

[107] Li C., Dong J., Han Z., Zhang K. (2017). MicroRNA-219-5p Represses the Proliferation, Migration, and Invasion of Gastric Cancer Cells by Targeting the LRH-1/Wnt/β-Catenin Signaling Pathway. Oncol. Res..

[108] Jian B., Li Z., Xiao D., He G., Bai L., Yang Q. (2016). Downregulation of microRNA-193-3p inhibits tumor proliferation migration and chemoresistance in human gastric cancer by regulating PTEN gene. Tumour Biol..

[109] Zhang X., Yan Z., Zhang J., Gong L., Li W., Cui J., Liu Y., Gao Z., Li J., Shen L., Lu Y. (2011). Combination of hsa-miR-375 and hsa-miR-142-5p as a predictor for recurrence risk in gastric cancer patients following surgical resection. Ann. Oncol..

[110] Chiang Y., Zhou X., Wang Z., Song Y., Liu Z., Zhao F., Zhu J., Xu H. (2012). Expression levels of microRNA-192 and -215 in gastric carcinoma. Pathol. Oncol. Res..

[111] Liu J., Ma L., Xu J., Liu C., Zhang J., Liu J., Chen R., Zhou Y. (2013). Spheroid body-forming cells in the human gastric cancer cell line MKN-45 possess cancer stem cell properties. Int. J. Oncol..

[112] Zhang X., Qian Y., Li F., Bei S., Li M., Feng L. (2018). microRNA-9 selectively targets LMX1A to promote gastric cancer cell progression. Biochem. Biophys. Res. Commun..

[113] Vlachos I.S., Zagganas K., Paraskevopoulou M.D., Georgakilas G., Karagkouni D., Vergoulis T., Dalamagas T., Hatzigeorgiou A.G. (2015). DIANA-miRPath v3.0: Deciphering microRNA function with experimental support. Nucleic Acids Res..

[114] R Development Core Team R: A Language and Environment for Statistical Computing. http://www.R-project.org.

[115] Reimers M., Carey V.J. (2006). Bioconductor: an open source framework for bioinformatics and computational biology. Methods Enzymol..

[116] Mishra L., Derynck R., Mishra B. (2005). Transforming Growth Factor-ß Signaling in Stem Cells and Cancer. Science.

[117] Han B., Cai H., Chen Y., Hu B., Luo H., Wu Y., Wu J. (2015). The role of TGFBI (βig-H3) in gastrointestinal tract tumorigenesis. Mol. Cancer.

[118] Choi Y.J., Kim N., Chang H. (2015). Helicobacter pylori-induced epithelial-mesenchymal transition, a potential role of gastric cancer initiation and an emergence of stem cells. Carcinogenesis.

[119] Nishimura K., Semba S., Aoyagi K., Sasaki H., Yokozaki H. (2012). Mesenchymal stem cells provide an advantageous tumor microenvironment for the restoration of cancer stem cells. Pathobiology.

[120] Donnelly J.M., Engevik A.C., Engevik M. (2014). Gastritis promotes an activated bone marrow-derived mesenchymal stem cell with a phenotype reminiscent of a cancer-promoting cell. Dig. Dis. Sci..

[121] Peng X., Liu G., Peng H., Chen A., Zha L., Wang Z. (2018). SOX4 contributes to TGF-β-induced epithelial–mesenchymal transition and stem cell characteristics of gastric cancer cells. Genes Dis..

[122] Zhou G.X., Li X.Y., Zhang Q., Zhao K., Zhang C.P., Xue C.H., Yang K., Tian Z.B. (2013). Effects of the hippo signaling pathway in human gastric cancer. Asian Pac. J. Cancer Prev..

[123] Qiao Y., Li T., Zheng S., Wang H. (2018). The Hippo pathway as a drug target in gastric cancer. Cancer Lett..

[124] Luo W., Brouwer C. (2013). Pathview: An R/Bioconductor package for pathway-based data integration and visualization. Bioinformatics.

[125] Abe Y., Minegishi T., Leung P.C. (2004). Activin receptor signaling. Growth Factors.

[126] Shi L., Resaul J., Owen S., Ye L., Jiang W.G. (2016). Clinical and Therapeutic Implications of Follistatin in Solid Tumours. Cancer Genom. Proteom..

[127] Mo J.S., Park H.W., Guan K.L. (2014). The Hippo signaling pathway in stem cell biology and cancer. EMBO Rep..

[128] Park J.H., Shin J.E., Park H.W. (2018). The Role of Hippo Pathway in Cancer Stem Cell Biology. Mol. Cells.

