# Supplementary material for: The Role of MicroRNAs in the Regulation of Gastric Cancer Stem Cells: A Meta-Analysis of the Current Status
Source: J Clin Med. 2019 May 9;8(5):639. doi: 10.3390/jcm8050639 (PMC6572052; doi:10.3390/jcm8050639)
Supplement: Supplementary file 1 [file jcm-08-00639-s001.pdf]

Supplementary Materials

The Role of MicroRNAs in the Regulation of Gastric Cancer Stem Cells: A Meta-Analysis of the Current Status

Vitalba Ruggieri [1], Sabino Russi [1], Pietro Zoppoli [1], Francesco La Rocca [1], Tiziana Angrisano [2], Geppino Falco [2,3], Giovanni Calice [1] and Simona Laurino [1,*]

[1] Laboratory of Preclinical and Translational Research, IRCCS-CROB, Referral Cancer Center of Basilicata, Rionero in Vulture, Italy. vitalba.ruggieri@crob.it (V.R.); sabino.russi@crob.it (S.R.); pietro.zoppoli@crob.it (P.Z.); francesco.larocca@crob.it (F.L.R.); giovanni.calice@crob.it (G.C.)

[2] Department of Biology, University of Naples Federico II, Naples, Italy. tiziana.angrisano@unina.it (T.A.)

[3] Section of Stem Cell and Development, Istituto di Ricerche Genetiche "Gaetano Salvatore" Biogem s.c. a.r.l., Ariano Irpino, Italy. geppino.falco@unina.it (G.F.)

* Correspondence: simona.laurino@crob.it (S.L.); Tel.: +39 0972 726239

Table S1. Significant KEGG pathway by predicted up regulated miRNAs.

KEGG pathway	*p-value*	N. of Genes	N. of miRNAs
Metabolism of xenobiotics by cytochrome P450	0	3	1
TGF-beta signaling pathway	3.96E-05	19	3
Estrogen signaling pathway	0.0002	16	3
Prolactin signaling pathway	0.0005	20	2
Axon guidance	0.0017	27	2
Lysine degradation	0.0023	7	1
Hippo signaling pathway	0.0080	19	1
Regulation of actin cytoskeleton	0.0177	32	1
Biotin metabolism	0.0261	1	1

P-value < 0.05 was considered as statistically significant.

Table S2. Significant KEGG pathway by predicted down regulated miRNAs.

KEGG pathway	*p-value*	N. of Genes	N. of miRNAs
Lysine degradation	0	14	10
Signaling pathways regulating pluripotency of stem cells	0	60	11
Estrogen signaling pathway	0	29	13
Proteoglycans in cancer	0	67	13
TGF-beta signaling pathway	0	36	16
Axon guidance	1.05E-08	39	11
Prion diseases	0.0001	3	1
Thyroid hormone signaling pathway	0.0004	21	2
Glioma	0.0012	26	8
MAPK signaling pathway	0.0019	65	6
Neurotrophin signaling pathway	0.0045	25	8
FoxO signaling pathway	0.0103	48	4
Prolactin signaling pathway	0.0193	20	7
Morphine addiction	0.0245	19	6
Hippo signaling pathway	0.0271	46	5
Regulation of actin cytoskeleton	0.0316	35	6
Pancreatic cancer	0.0387	10	2
Chronic myeloid leukemia	0.0475	22	3

P-value < 0.05 was considered as statistically significant.

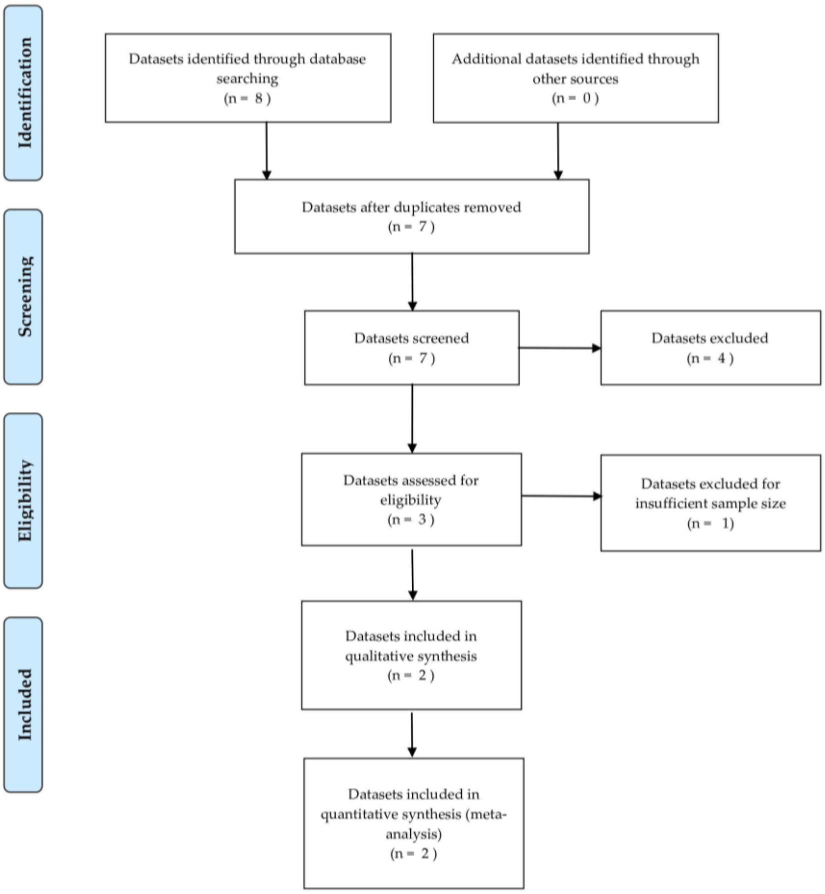

Figure S1. PRISMA (Preferred Reporting Items for Systemic Reviews and Meta-Analyses) flow diagram.
